# Parental experience of the neuromotor development of children with congenital heart disease: an exploratory qualitative study

**DOI:** 10.1186/s12887-021-02808-8

**Published:** 2021-10-01

**Authors:** Elena Mitteregger, Martina Wehrli, Manuela Theiler, Jana Logoteta, Irina Nast, Brigitte Seliner, Beatrice Latal

**Affiliations:** 1 Child Development Center, University Children’s Hospital Zurich, Steinwiesstrasse 75, CH-8032 Zürich, Switzerland; 2 Children’s Research Center, University Children’s Hospital Zurich, Zurich, Switzerland; 3 School of Health Professions, Zurich University of Applied Sciences, Winterthur, Switzerland; 4 Faculty of Medicine, University of Zurich, Zurich, Switzerland; 5 Swiss parents’ association for the child with heart disease (Elternvereinigung für das herzkranke Kind), Mülligen, Switzerland; 6 Department of Pediatric Cardiology, University Children’s Hospital Zurich, Zurich, Switzerland; 7 Swiss Children’s Rehab, University Children’s Hospital Zurich, Zurich, Switzerland

**Keywords:** Congenital heart disease, open heart surgery, infant, child, parental experiences, motor development delay, burdens, concerns, qualitative study

## Abstract

**Background:**

Children with severe congenital heart disease (CHD) are a group of children at risk for neurodevelopmental impairments. Motor development is the first domain to show a delay during the first year of life and may significantly contribute to parental concerns, stress, and difficulties in early child-parent attachment. Thus, the aim of the study was to better understand the wishes and concerns of parents of children with CHD and explore their experience of their children’s neuromotor development in the first year of life.

**Methods:**

In this qualitative study, fourteen families were recruited. Their children were aged 1–3 years and had undergone open heart surgery within the first 6 months of life. Semi-structured interviews were audio-recorded and transcribed. The data was explored within an expert group, and a qualitative content analysis was conducted using VERBI MAXQDA software 2020. The study was conducted in accordance with the COREQ checklist.

**Results:**

Parents of children with CHD reported several burdens and needs. Parental burdens concerned the child’s motor development, their own physical and psychological strain, and difficulties in communication with healthcare professionals. The needs, parents reported included supporting their child’s motor development, a medical coordinator, and better communication between healthcare professionals and parents. During the first phase of their children’s illness, parents underwent a dynamic transitional phase and expressed the need to rely on themselves, to trust their children’s abilities, and to regain self-determination in order to strengthen their self-confidence.

**Conclusions:**

It is essential to involve parents of children with CHD at an early stage of decision-making. Parents are experts in their children and appreciate medical information provided by healthcare professionals. Interprofessional teamwork, partnering with parents, and continuous support are crucial to providing the best possible care for children and their families. Family-centred early motor intervention for CHD children might counteract the effect of parental overprotection and improve children’s motor development and thus strengthen child-parent interaction. In future work, we aim to evaluate a family-centred early motor intervention for children with CHD developed on the basis of this qualitative study.

**Trial registration:**

Not applicable.

**Supplementary Information:**

The online version contains supplementary material available at 10.1186/s12887-021-02808-8.

## Background

Congenital heart disease (CHD) constitutes the most common congenital malformation in newborns, occurring in approximately 8 of 1000 live-born children [1, 2]. Improvements in surgical intervention and perioperative care have increased the survival rate dramatically in recent decades to its current level of around 90 %, even for the most severe forms of CHD [3, 4]. In contrast to this significant achievement, children are at high risk of neurodevelopmental disorders that manifest preoperatively [5]. Developmental abnormalities may be related to delayed brain maturation [6, 7] and to perioperative white matter injuries occurring in 30–50 % of all newborns with CHD [8]. This seems to correlate with worse school-age neurodevelopment in children with critical CHD [9]. Motor development is the first developmental domain to be affected with a prevalence of 40–60 % within the first year of life, followed by other developmental impairments such as language disorders and behavioural and learning difficulties, which occur later at school age [10].

Neurodevelopmental impairments can be ameliorated by early interventions. Researchers like Cioni and colleagues and Guzzetta [11, 12] have summarized which components are essential to early interventions’ maximally effective prevention of maladaptive plasticity in infants’ brains: It has to start early; should be intense, active, and tailored for each individual; and must involve children and their family. However, it remains unclear which early motor intervention is optimal for children with CHD. Involving families in care and therapy has become more prominent in recent decades [13, 14] but only seems to be partially implemented for children with CHD and their families. This is particularly important because parents of children with CHD face a variety of stress factors related to the disease that influence parental quality of life and their children’s development [15–20].

However, what do we actually know about the parents’ wishes and concerns about early motor intervention for a child with CHD? To the best of our knowledge, no study has yet addressed this question. We therefore formulated our research question: How do parents experience their children’s motor development during the first year of life after open- heart surgery for CHD? Our qualitative study also pursued the aim of tailoring an early motor intervention trial for these children.

## Methods

### Study design

This study used an exploratory qualitative design using individual interviews applied as described by Sandelowski [21, 22] and Neergard [23].

This qualitative study serves as a basis on which we develop a family-centred early motor intervention for children with CHD after open-heart surgery, incorporating parental experiences and needs. In a second step we will investigate the feasibility and effectiveness for children with CHD of this intervention.

This study is part of a PhD project led by EM, a senior paediatric physiotherapist. Her extensive experience in early intervention with infants at risk resulted in thorough investigation of the motor development of children with CHD.

This study was performed in accordance with the Declaration of Helsinki [24]. The Ethics Committee of the Canton of Zurich confirmed the correctness of the procedure of this project (BASEC-Nr. Req-2019-00517). All parents gave written consent and acknowledged that they would not be identified in this paper because all names were removed to protect anonymity.

### Recruitment and enrolment

Parents of children with CHD who had undergone open heart surgery within the first 6 months of life were recruited via an advertisement in the journal of the Swiss parents’ association for the child with heart disease (Elternvereinigung für das herzkranke Kind). Two more families were referred to us by a physiotherapist. One family was known to EM. Their child was referred to physiotherapy for another indication than CHD. No other family had any prior connection to the author. Study information was provided to all participants, and the purpose of the study was explained. Parents needed to have an adequate understanding of the German or English language to be included. Fifteen families contacted us; one family had to be excluded because the child was younger than one year of age, and all others were included consecutively in this study.

### Data collection

After consent had been obtained, parents completed a family demographic questionnaire that collected information such as living and work situation, their mother tongues, income, and education level with Research Electronic Data Capture (REDCap) electronic data capture tools hosted at the University Children’s Hospital Zurich [25, 26]. REDCap is a secure, web-based software platform designed to support data capture for research studies. Additional data were collected using individual, semi-structured interviews. All interviews were conducted by EM and lasted from 22 to 54 min in length, with a mean of 33 min.

Nine interviews were carried out at the families’ homes, two at parental working places and two at the Children’s Hospital. One interview was carried out with two participating families (families 6 and 8) present for organizational reasons. Ten interviews were attended by mothers only, three were conducted with both parents present, and in eleven the children with CHD were present. All interviews were carried out using an interview guideline (see supplementary material I) and were audio-recorded between October 2019 and March 2020. Prior to data collection, the interview guideline was pilot-tested. The guideline tackled four key themes: (a) parental experience of the period at home after surgery, (b) the child’s development in the first year of life, (c) the support that parents had received during that period, and (d) one open topic that parents wanted to talk about before finishing the interview. After each interview, field notes were written to document details about the atmosphere, the interaction with the interviewees, and the length of the interview (see supplementary material II).

### Participants

We included parents of children with CHD, after open heart surgery within the first 6 months of life, whose children were between 1 and 3 years of age before the interviews were conducted. Whereas the interviews with a single parent allowed an exploration of parents’ individual experiences, the parents who were interviewed together gave a picture of the commonalities and differences in both their experiences and their coping strategies. We used a nonprobabilistic, purposive sampling, which was sufficient enough to gain information and reach saturation [27] and [28].

### Data analysis

Data were analysed using qualitative content analysis based on Elo and Kyngaes [29] and Schreier [30] and were reported according to the COREQ checklist [31]. Data were coded inductively and inclusively to ensure that context was preserved. Interviews were transcribed by IR, SM, and EM. Prior to coding, all transcripts were compared to the audio files and proofread by MB to check for accuracy, and all families were offered the opportunity to add comments and corrections to the transcripts. Four families took up this offer. No changes were suggested. The content of all interviews was dissected using VERBI Software MAXQDA 2020 [32], a computer-based analysing program.

Preparatory to the analysis, EM (MScPT, paediatric physiotherapist, researcher) inductively coded the content of the first four transcripts independently prior to creating an initial set of categories with in vivo codes. Thereafter, MW (MScPT, physiotherapist, researcher) was given the codes and the first four interview transcripts for better understanding and created her own set of categories. EM and MW discussed their sets of categories and adjusted and rearranged them until consensus was found. After this process, the rest of the interviews were coded by EM. In the analysis phase, EM and MW grouped the in vivo codes of all interviews individually into subcategories before reviewing the codes, categories, and subcategories together. No new aspects emerged, saturation of data was attained in accordance with qualitative research. Disagreements were discussed until consensus was found. The category system was further modified and condensed, and themes were developed in an iterative process within an interdisciplinary, all-female team, consisting of EM, MW, BS (PhD RN, paediatric advanced practice nurse), IN (Prof Dr, psychologist, researcher), and BL (Prof MD, MPH, paediatrician, researcher). All the researchers have extensive experience in paediatrics and/or qualitative research. The abstraction process led from in vivo codes via subcategories to categories and ended in the generation of themes. Examples of this process are found in the supplementary material III. Parental quotations were translated from German into English by a professional translator.

### Measures of trustworthiness

In order to check for trustworthiness, we used the criteria of credibility, dependability, confirmability, and transferability described by Elo and Kyngaes [29], Graneheim and Lundman [33], and Neergard [23]. Variation in sampling provided a broad insight into participants’ perceptions, which provided credibility. The participating parents exhibited a bread variation in socioeconomic status as expressed by educational level and occupation. Parents were encouraged to speak openly using open-ended questions. If responses were not clear additional questions were asked. A trial interview was performed to test applicability, the process of data collection was recorded, and a description of the analysis was collected in a log file.

The same interview guideline was used for all interviews, and all parents were interviewed by EM, which provided dependability. Confirmability was provided by handling all interview data systematically, by repeated readings to capture parental expert knowledge, and by requesting parental feedback on the preliminary results. The categories were illustrated with interview quotations. Consensus was reached within the research team, to ensure that participants’ data were correctly displayed. Final results were presented to MT (MD, mother of a child with CHD, representative of the parents’ association of children with CHD) and discussed to ensure transferability. This was further promoted by presenting participants’ voices trustworthily using explicit parental quotations.

A summary of the preliminary results was sent to all interviewees via REDCap [25, 26] for parental checking.

## Results

Seventeen parents (14 mothers and 3 fathers) completed the family demographic questionnaire (Table 1); other characteristics such as parental age, working hours, child’s age at the time of the interviews, and location of the interviews are provided in Tables 1 and 2. Nine interviews were conducted in Swiss German, four in German and one in English. Ten of 14 children with CHD were girls.

**Table 1:** Sociodemographic characteristics

At the time of interview	Median (min; max)
age mother [years]	35.5 (30; 44)
age father [years]	37.0 (25; 49)
no. of children per family	1 (1; 3)
age of child [months]	23.5 (12; 41)
working hours mother [% FTE]^a^	45 (0; 100)
working hours father [% FTE]	100 (80; 100)

^a^ FTE full-time equivalent, expressed as percentage of a full working week; a full working week is equivalent to 42 hours in Switzerland

**Table 2:** Description of interviewed families

Family Number	Number of children	Child’s age at interview [months]	Interviewee	Place of interview
1	2	24	Mother	home
2	1	17	Mother and Father	home
3	1	41	Mother	workplace
4	1	18	Mother and Father	home
5	2	18	Mother	home
6^a^	1	16	Mother	workplace
7	2	24	Mother	home
8^a^	1	36	Mother	workplace
9	2	24	Mother	Children’s Hospital
10	2	23	Mother	home
11	1	24	Mother	home
12	1	12	Mother and Father	home
13	1	16	Mother	home
14	3	36	Mother	Children’s Hospital
	21	Mean age 23.5 (12; 41)	Mother: 11 Father: 0 Both: 3	Workplace: 3 Children’s Hospital: 2 At home: 9

^a^ mothers of families 6 and 8 participated together in a single interview

Overall, 86 % of the participants felt themselves well represented in the preliminary results (score from 0 [not at all] to 10 [totally]). Some respondents did not feel well represented because they felt they that had been supported by medical staff members and were satisfied with the rehabilitation process of their infant after heart surgery.

**Fig. 1:**
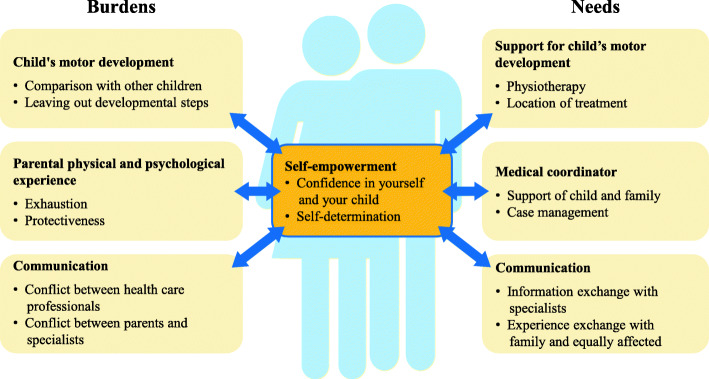
Range of generated themes of the interviews

The main themes that parents of CHD children reported were the **burdens** and **needs** they had experienced (see Fig. 1). During the first year of their children’s illness, parents went through a dynamic transitional phase and expressed the need to develop self-empowerment. Results are described in detail below.

### Burdens

Parents were challenged by their child’s heart defect at various levels. Burdens crystallized into three categories. These were the child’s motor development, the parents’ physical and psychological experience, and the problematic area of communication, which here included both communication between disciplines and communication between professionals and parents.

#### Child’s motor development

Their children’s motor development after open-heart surgery worried almost all parents of children with CHD. They often reported abnormalities in their child’s motor development during the first year of life. Comparisons with other children and the absence of developmental steps were often mentioned.

##### Comparison with other children

Parents observed their children and compared them with other children of the same age in their surroundings. Most parents noticed that their children developed more slowly than healthy children. This unsettled the parents and led them to oscillate between hope and discouragement, as the following quotes show:

*Yes, at the beginning I had a hard time comparing with my friend, whose child is three months older, and I thought at the beginning, oh no, he’s already right behind.* [family 5]

*You always compare a bit. And you think, yes, children who are a year younger can already walk, and he still can’t. … we just have to give him time, don’t we? … Then you just get kind of insecure.* [family 10]

*Comparing my child with your child doesn’t help much, of course; actually, it can be very discouraging.* [family 11]

*We’ve also seen children in the Children’s Hospital who are much older and can’t do anything like as much.* [family 4]

*If you see a child with the same condition and you see her running around that means my child is going to be good. It gives you hope.* [family 9]

##### Leaving out developmental steps

Almost half of the parents said that their child did not tolerate lying in prone position and in some cases, also skipped crawling, and they attributed this to heart surgery.

*I don’t know but she hated that, she always cried when you put her on her tummy, so we were not even able to do all of that.* [family 9]

*I noticed it quite early on, because lying her on her stomach was not possible…* [family 3].

*He always bottom-shuffles now; he never crawled.* [family 12]

#### Parental physical and psychological experience

All parents felt relieved when they returned home to their familiar surroundings, but for many of them, assuming sole responsibility for medical care and the coordination of appointments for their child was a physical and psychological burden. Parents reported on their need to take more care of their child.

##### Exhaustion

Frequent medical and therapeutic appointments were time consuming and demanding and required great flexibility from parents. The care of children with CHD was a challenge for the parents. Many parents reported these additional demands on them, as the following quotations show:

*There were about three or four appointments per week … then you have to somehow arrange each day around various appointments. And that was just really difficult… you’re always out of the house for at least three hours.* [family 13]

*It gets tiring. It is already tiring to have a baby, and then it is even more tiring to have a baby with needs.* [family 9]

*Because her oxygen level was very low, and I had to watch her on a monitor and … decide, do I have to contact you now or can I wait another night*? [family 1]

*You are very exposed, and you know that you can’t really plan anything, you can’t organize anything, you can’t plan anything.* [family 6]

##### Protectiveness

Parents reported that they perceived their child with CHD to be vulnerable and protected the child from overexertion and avoidable dangers such as falling. The constant monitoring of vital signs and the fear and concern for their child during everyday life increased their emotional strain, as these quotations from families show:

*It’s always in your mind: you never know if your child will shut down in the next half hour or not … all night the light was on so that I could see when I woke up if she was still moving, still breathing.* [family 3]

*He was, like, more fragile than others. He probably wasn’t really, but we all treated him like that … we treated him more carefully, I feel.* [family 10]

*Just don’t fall, and don’t get bruised, and please be careful; come here, I’ll carry you up the stairs – so she doesn’t have to walk, because that could easily mean blue lips, and even slightly blue lips mean maybe the heart won’t work.* [family 14]

In addition, in a few families the extraordinary situation posed a challenge to the couple’s relationship. For some families, professional and financial worries increased the burden. Parents who sought help from their family reported a reduction in their workload.

#### Communication

Parents reported how challenging it was to be in the problematic field between specialists and other professionals. In addition, parents felt obliged to mediate between the various specialists. The large number of people involved who were responsible for their child also made communication more difficult.

##### Conflict between healthcare professionals

Conflicting opinions from medically trained specialists and other professionals contributed to the feeling of insecurity among some parents. It was difficult for parents to deal with the fact that, for example, the specialist and the paediatrician had different opinions.

*So the cardiologists have their opinion, and the paediatrician has his opinion. [family 10] *

*What was so overwhelming was when someone came and gave me a tip…. But I thought that this contradicted the advice of the previous nurse. … I want to do everything right, so what do I do now?* [family 12]

##### Conflict between parents and specialists

Parents reported that they had to take on the role of mediator between professionals in order to ensure optimal care for their child. Sometimes, it was also unclear who had responsibility for the care of their child.

*In between I feel as if I am … a specialist … first I have to go to the I. [paediatric heart centre], then I have to tell the paediatrician that everything’s fine.* [family 3]

*We’ve seen thousands of people again and again about the same thing and it’s just kind of complicated for everyone. The people there don’t know their way around …*. [family 13]

### Needs

The results reflect three aspects of parents’ needs: support for the child’s motor development, the need for a medical coordinator, and communication between all those involved.

#### Support for child’s motor development

Regular support from physiotherapy enabled parents to have a valuable exchange with a developmental specialist who kept track of their child’s motor development. This gave the parents security and support about their child’s development.

##### Physiotherapy

Parents appreciated the holistic approach of physiotherapy and the suggestions and ideas for playful implementation in everyday life. The involvement of parents and siblings was felt to be valuable and supportive.

*If she [the physiotherapist] says something, then I really have to look at home; I have to make this and that possible for him, maybe just so that he can climb up something. Or she shows me what we can do at home.* [family 10]

*How to learn how to sit up, how to learn how to stand up, how to do this or that, how to support the child. Such detailed things have already helped me, you know, I didn’t even know about them.* [family 1]

*And she [the physiotherapist] also integrated our daughter and gave her tasks to do with him (brother with CHD) at home.* [family 5]

##### Location of treatment

Where the physiotherapeutic treatment takes place was perceived differently depending on the family situation. Some experienced the external appointment as a change, others found the familiar surroundings at home helpful, as the following quotations show:

*So doing this at home wasn’t an option for us, because I think it’s important to get away from everyday life and be able to keep appointments. That gives you a break, it gives you a change, and it’s good for everyone.* [family 11]

*That would certainly be helpful. I mean, home is a familiar environment, and she reacts differently, so if she first experiences these things at home …* [family 13].

The needs of parents whose child did not receive physiotherapy are almost identical to the experience of families who did.

*It would have been good for me if I had had therapy for a while afterwards, simply because of the social contacts with specialists…. You can also exchange ideas and say yes, it’s normal … what can I do at home to support her…* [family 3].

#### Medical coordinator

Parents wanted both someone who would constantly accompany them in the long term and someone who would organize or take over the coordination of the many medical appointments.

##### Support of the child and family

The quotation below provides an example of the parents’ often expressed wish for someone who knows the history of the child and the family.

*I [a mother of two children with CHD] think that physiotherapy is very important, especially during the first year of life … I have already found this accompaniment very valuable with both children* [family 1].

##### Case management

The desire for someone who would be able to connect to a heart centre and ensure the coordination of appointments and the flow of information for all involved was expressed repeatedly.

*That there is someone who looks at all the factors and then can inform the bodies that need it or set something in motion. For us, these are always just separate pieces of the puzzle.* [family 10]

*That you aren’t going to be forgotten at home … that when you leave [the hospital], someone makes sure that you’re still well connected.* [family 7]

*I really think cooperation and a better organization … it’d be great to have someone who can take care of everything* [family 13].

#### Communication

Parents expressed the need to receive comprehensible information from specialists. They felt that the exchange of experience with family members and people with the same concerns provided important support in overcoming the challenge.

##### Information exchange with specialists

The desire for a better explanation of the diagnosis and further treatment was mentioned by many parents. Parents suggested several times that medical staff should ask for their expertise.

*What’s still important to me, I’ve come to realize, is that one needs to become more deeply enlightened…. Sure, you don’t have to scare the parents. But the parents will inform themselves anyway* [family 10].

*I also got some literature before. I’m the kind of person who wants to know what’s done with my child … how a heart-lung machine works. … If I know how it works, even roughly, and find it logical, then it helps me keep calm.* [family 6]

*A doctor who responds to you as a parent, who doesn’t see your questions as ridiculous or nonsensical – you can see that in their faces sometimes – but really takes them seriously, maybe thinks about them again and then gives you another answer.* [family 12]

*They didn’t respond to us. We as parents know better what she does and doesn’t do, and that they didn’t really respect that.* [family 2]

##### Experience exchange with family and equally affected

Parents repeatedly described how important the exchange with family and equally affected others was in coping with the challenge of caring for their child. Accepting or requesting support from their own families in good time proved to be helpful.

*We should certainly have accepted more help, but not from the hospital, from specialists, but rather from the family.* [family 5]

*Communication with each other is important, with the partner or with the family, also talking about it … and the exchange with other heart children, somehow, we were glad we had people who were in the same situation.* [family 4]

*What would have been good for during this time would have been an environment of parents and mothers who had been through the same thing. Of course, everyone’s story is individual, but where we could exchange experiences.* [family 8]

### Self-empowerment

Parents went through a process with their child’s heart disease and, looking back, drew lessons from it. This process varied from family to family and also depended on personal experience, for instance the number of children in the family. During the first year of their children’s illness, parents went through a dynamic transitional phase and expressed the need to strengthen their self-confidence.

#### Confidence in yourself and your child

Parents described how the diagnosis shook their confidence in themselves and their child. They experienced great insecurity:

*Because it shakes you at the first moment when you get the diagnosis. It shakes everything up…. Yes, what does that actually do to me, the issue of trust or basic trust, even in relation to your child.* [family 3]

The importance of regaining confidence in this process, relying on one’s intuition and so strengthening oneself, allowing the child to have their own experience and trusting them to do so, was often given as a suggestion. In addition, the parents advised others of the same age to follow the path they considered to be the right one.

They described it as follows:

*I think we trusted ourselves, our intuition.* [family 4]

*… that you take the child the way she is … she simply develops for herself and at her own pace, in her own way* [family 12].

*Pushing the child out of the nest early … having the confidence that other people could do the same with her even with the heart defect.* [family 14]

*I think she’s a perfectly normal child that also has a heart defect and not that she is the child with a heart defect … we go to the playground, we just let her do it. She tries and learns on her own; we are not so afraid that she might fall down.* [family 7]

#### Self-determination

Parents reaffirmed the importance of taking the path that they considered right in their child’s care and attention, perhaps even despite well-meant advice or opinions from others.

*And do what they [the parents] want. That is something very important, doing it the way they want, regardless of what anyone thinks.* [family 14]

*Yes, you can tell me things, but then we’ll discuss them together, and decide how we’ll go on. And this is the path we’ve chosen. And we didn’t let this shake us anymore.* [family 12]

*Above all … all the advice that everyone gives you – you have to do it this way, you have to do it this way – they all mean well, but they’re … just wrong.* [family 13]

*The child comes back, he is doing well so far. They say ‘yes it will be alright,’ ‘everything’s going to be fine.’ They mean well, but they haven’t got a clue.* [family 10]

## Discussion

The aim of this study was to understand more about the wishes and concerns of parents with children with CHD after open-heart surgery and to explore their experience of their children’s neuromotor development during the first year of life. In our study, we identified specific burdens and needs. Parental burdens included the child’s motor development, their own physical and psychological strain, and difficulties in communication with healthcare professionals. The needs that parents reported included supporting their children’s motor development, medical coordinators, and better communication between healthcare professionals and parents. Parents highlighted the importance of self-empowerment during the first period of their children’s illness.

The lives of parents are shaken to their foundations when their children are diagnosed with complex CHD requiring cardiac surgery. Parents included in our study experienced loss in confidence in themselves and their children. These findings coincide with results by many other researchers reporting that parents of children with complex CHD undergo intense emotional stress, anxiety, and perceive hopelessness [15, 34–37].

The hospital setting with its own rather inflexible and hierarchical structures and measures vital for their children’s survival cause parents to lose their self-determination. Parents have to place their children’s fate into the hands of unknown people. With that, parents’ roles as their children’s central guardians become those of helpless observers [37]. Parents use all their resources to be there for their children during the hospital stay, on top of their work and other commitments such as the care of siblings. The burden of care for such families leads to emotional costs experienced by these parents. Connor et al. [38] described both, life-change and uncertainty. Compared to their study, in which many parents also indicated financial strains associated with their child’s disease, only two families in our study group indicated that they had experienced financial problems. This might have to do with generally higher salaries and well-established unemployment insurance in Switzerland. However, in our opinion this situation may change over time if the parent of a chronically ill child gives up work to care for the child.

When children are discharged after surgery, families are presented with new challenges. They are on their own without medical supervision and need to take over their children’s medical care without being healthcare professionals. In their mixed-methods study, Gramszlo et al. [20] included parents of children with CHD of 1–3 years after surgery before 6 months of age. They reported about the ongoing influence of uncertainty and challenges after discharge on parents. In a qualitative study, Rempel et al. [34] interviewed parents and grandparents of 15 young children (6 months–4.5 years) who had undergone heart surgery. These researchers investigated the phases of parenting under pressure and described how parents encountered new challenges during transitions between hospital and home.

More time together with their children at home than in the hospital setting allows parents to experience and observe their children more precisely. Parents then might detect differences between their children’s development and that of healthy children, which can further increase parental stress and reduce confidence in their children’s ability.

There is a large body of evidence that children with CHD often present with delayed motor development that is associated with generalized muscular hypotonia [39, 40]. Infants with muscular hypotonia often dislike prone or four-point kneeling because these positions require adapted muscle activity against gravity. These children seek for other movement strategies and often prefer bottom shuffling. Muscular hypotonia might be why bottom-shuffling children seem to start walking later than those crawling on hands and knees, as described by Størvold et al. [41]. Strikingly, many parents in our study reported that their CHD children avoided active prone or crawling on hands and knees.

Parents of children with CHD after open heart surgery seem to be reluctant to challenge their children and to protect them as much as possible and stress them as little as possible. Although this reaction is very understandable, one could argue that parents tend to overprotect their CHD children, which might lead to short and longer-term developmental risks: Could motor delay in CHD children be partially due to parents’ overprotectiveness and their fear of over-straining their children during physical activities? Majnemer et al. [42] and Williams et al. [15] support this assumption. Our participating parents reported that they tended to handle their children very carefully, to avoid uncomfortable positions such as prone, and to watch their children constantly. This was also noted by Rempel et al. [34]. Other researchers have described how the long-term effect of parental overprotection may lead to heart-focused anxiety in adolescents and adults with CHD [43, 44].

The combination of low muscle tone and parental overprotectiveness may further hamper CHD children’s motor development. It seems more than possible that these children do not leave their comfort zone easily and thus deprive themselves of the opportunity to promote their own motor development and the exploration of their environment intensively from early infancy on. There is evidence that self-produced sensorimotor experience plays a pivotal role in motor development [45]. A reduction in physical activity that starts very early most likely continues during childhood and later. According to a nationwide survey in Germany by Siaplaouras et al. [46], physical activity is markedly reduced in children with CHD. The results suggest that as well as overprotection by caregivers, teachers, and trainers, physicians and healthcare professionals also tend to overprotect these children and recommend restrictions on levels of physical activity.

Motor developmental delay is the first developmental problem to become apparent within the first year of life in CHD children [10]. Thus, early intervention can provide a window of opportunity to prevent or treat neuromotor delay, strengthen child-parental interaction, and thus promote healthy overall development. It has been shown that factors including positive relationship with caregivers and self-efficacy promote resilience in early life [47].

Paediatric physiotherapists are specialists in early motor development. They empower parents by teaching them to mindfully observe and understand their children’s development. Physiotherapists encourage the children’s activity and thus support children and parents alike to move beyond their comfort zones. They encourage parents to let their children explore their bodies and environments and promote the children’s development. Physiotherapists often become figures of trust and reference as they monitor children’s development on a regular basis. Families in our study whose children had received physiotherapy felt well looked after. Also, parents stated that the location where the physiotherapy should take place, either in an outpatient setting, at home, or as a mixture of both, should be decided individually by each family.

In our interviews, the essential topic of communication and associated difficulties crystallized as an extensive theme. Parents reported communication to be an area of conflict both within the healthcare teams and between themselves and specialists. Generally, parents value medical details and report the importance of in-depth information provided by healthcare teams for them to learn as much as they can about their children’s medical condition, treatment, and ways to optimize their children’s outcome [15, 34]. This corresponds to the need for more information exchange referred to by our participating parents.

Additionally, the wish for a medical coordinator was expressed by many participants in our study. Parents wanted someone who knows the history of the child and the family and who monitors development in the first year of life. Moreover, they looked for a person that keeps all the ‘pieces of the puzzle together’, overviews appointments and connects healthcare professionals. However, this communication seems to be insufficient or lacking altogether. Parents report that they often need to adopt the role of a coordinator by managing appointments and exchanging information between the healthcare teams. This might serve as an additional cause for exhaustion. The importance of parental support, the confusion about parents’ roles and the problem of information transparency has been well described previously [15, 20, 48]. Realistically, the role of a medical coordinator cannot be fulfilled by a single person. We think that the role of supporting the family can be taken over by any member of the medical care team (e.g., physiotherapist, specialist, professional nurse, paediatrician) with the aim of unburdening the family.

Moreover, participating parents highlighted how important they considered and how much they appreciated their families’ and friends’ support in coping with the challenges presented by CHD. Parents indicated that exchanging experience with equally affected parents and receiving information and tips helped them to cope better with their children’s illness. Our results support those by Uzark and Jones [16], Sjostrom-Strand et al. [35] and McCusker et al. [49], who all stated the importance of social support for the well-being of CHD parents. One might consider that first-time parents need more support than other families as they cannot yet rely on previous experience. Of course, this applies to all first-time parents, but here we must emphasize the major difference that these parents additionally have to care for children with a chronic disease.

In our interviews, several parents advised equally affected caregivers to go their own way despite what others may think or say. This of course has to be treated with caution because not all parents might be aware of all medical consequences.

Parents expressed the need to rely on themselves, trust their children’s abilities, and retrieve self-determination after the initial period of their children’s illness. These factors enable them to regain their strength. Parents become experts in their own children with time and might then have more resources available in order to meet the challenges of their children’s disease. They need first to undergo certain experiences before being able to perceive what they need. This is another reason why it is even more essential to make sure that parents and the healthcare team work together as equals.

### Limitations

Our study has several limitations. Our results are based on interviews with parents who were willing to participate in our study and to invest their time in interviews. These parents may represent a group with particularly difficult or burdensome experiences. We cannot rule out recall bias, an issue in self-reporting past events that increases with time. Nonetheless, we tried to minimize this issue. We only included children that had undergone open heart surgery within the first 6 months of life and that were between 1 and 3 years of age at the time of the interviews.

We are also aware that the results of the interviews mainly represent the views of mothers. In only three interviews did both parents participate, and none of the interviews was conducted only with a father. Nonetheless, it has to be said that in general mothers are still more involved in children’s early life and carry the main burden of care. This in turn leads to a lower employment rate for mothers. This can be seen in our sample, in which mothers worked an average of 39 %, whereas fathers worked 97 %. Additionally, mothers of CHD children need to manage the medical care and meet the regular appointments that are necessary for their children.

We primarily focused on children with CHD and not on genetic comorbidities. At the beginning of their lives, the important focus is on their heart. Parents of children with another medical condition in addition to the CHD most likely face more difficulties [17].

## Conclusions

Our study underpins how essential it is to involve parents of CHD children in decision-making about the care of their children. Interprofessional teamwork, transparent communication, partnering with parents and continuous monitoring is crucial to provide the best possible outcome for children and their families. Parents appreciate medical information to better understand and support their children’s development from an early stage.

Taking into consideration that motor developmental delay is the first developmental problem to become apparent in CHD children, it is evident that physiotherapists specialized in child development, are best equipped to support and monitor these children and their families. An early motor intervention for CHD children could counteract the effect of parental overprotection, improve the children’s motor development and self-efficacy, and strengthen child-parental interaction.

In future work, we aim to evaluate a family-centred early motor intervention for children with CHD, developed on the basis of this qualitative study.

## Supplementary Information

**Additional file 1.** Interview guideline.

**Additional file 2.** Interview minutes.

**Additional file 3.** Examples of the methodological generation of themes.

## Data Availability

The datasets used and analysed in this study are available from the corresponding author on reasonable request.

## Abbreviations

CHD: congenital heart disease
BL: Bea Latal
BS: Brigitte Seliner
EM: Elena Mitteregger
IR: Irene Reize
IN: Irina Nast
JL: Jana Logoteta
MT: Manuela Teiler
MW: Martina Wehrli
SM: Susanne Margaris
