# Supplementary material for: Parental experience of the neuromotor development of children with congenital heart disease: an exploratory qualitative study
Source: BMC Pediatr. 2021 Oct 1;21:430. doi: 10.1186/s12887-021-02808-8 (PMC8485514; doi:10.1186/s12887-021-02808-8)
Supplement: Supplementary file 1 — Additional file 1. Interview guideline. [file 12887_2021_2808_MOESM1_ESM.docx]

| **Supplementary material I:**  **Interview guideline** | | | |
| --- | --- | --- | --- |
| **Key questions**  (Invitation to narrate) | **Check** (has this been mentioned)  **Memo** (ask, if not mentioned) | **Specific questions**  (at the appropriate time or at the end) | **Themes** |
| ***part I – at home after surgery***  *When you think back to the time when you came home with your baby after heart surgery, how was that for you?* | - Experiences, information about the hospital stay - Query previous experiences | *How did you feel about handling your child? What kept you busy then?*  *How did getting to know each other at home, work out?*  *Could you read the signals of your baby and respond to them? What gave you security and support?* | **Personal experiences**  **Parent-child interaction**  **Empowerment** |
| ***part II – child’s development***  *How did your child develop?*  *How did you perceive, see and experience the movements of your child?*  *Did you notice anything in your child’s development that was different to other infants?*  *How did this become apparent?* | - Memory, experience - Child’s behaviour: how did it express itself, how did it move, develop? - How did the development, the movements look like compared to infants of the same age?   Did you notice anything? | *What was it like for you when your child started playing with his body, started moving around?*  *In which situations did you notice this?*  *What observations did you make?* | **Experiencing child’s development**  **Comparison to other children** |

| ***part III – support***  *What do you think could have helped you then in your daily life?*  *What gave you security or support?*  *What offer would you make a family, today after heart surgery, what concrete offer would you make to this family to support them in the first year of life of their child?* | - Support, an ear - Information, instruction, coaching - Professional competence - Everyday situations - Concrete facts | *Have you sought assistance? Which ones (early education, Osteopathy craniosacral therapy, physiotherapy, grandma...)?*  *What did they contribute that others could not?*  *Who and what things could have supported you?*  *What if you and your child had been supported in the 1st year of your child’s life – what would you had preferred - support, therapy at home or at the hospital, outpatient clinic?* | **Need for**  **Support**  **Empowerment**  **Conclusion and perspective for the parents** |
| --- | --- | --- | --- |
| ***part IV – ending***  *Is there a topic that was not addressed and is still important to you?* | Closure/ending |  | **Review/outlook** |
