# Supplementary material for: Parental experience of the neuromotor development of children with congenital heart disease: an exploratory qualitative study
Source: BMC Pediatr. 2021 Oct 1;21:430. doi: 10.1186/s12887-021-02808-8 (PMC8485514; doi:10.1186/s12887-021-02808-8)
Supplement: Supplementary file 2 — Additional file 2. Interview minutes. [file 12887_2021_2808_MOESM2_ESM.docx]

**Supplementary material II**

**Interview minutes**

**ID-Code: __________________**

| **Interviewer** | name: _________________________________________________ |
| --- | --- |
| **Environment** | date: _________________ duration: ___________________min  place/facilities: |
| **Interviewee** | ☐ mother ☐ father ☐____________________________  age: ______________________________________________________  education: _________________________________________________  occupation: ________________________________________________  children: ___________________________________________________  family status: _______________________________________________ |
| **Motivation for participation** | __________________________________________________________  __________________________________________________________  __________________________________________________________  __________________________________________________________ |
| **Additional information**  (special occurrences during contact or interview) | __________________________________________________________  __________________________________________________________  __________________________________________________________  __________________________________________________________  __________________________________________________________ |
| **ID-Code:** | |
| **Atmosphere of interview** (key words on personal atmosphere) | ___________________________________________________________  ___________________________________________________________  ___________________________________________________________  ___________________________________________________________  ___________________________________________________________  ___________________________________________________________ |
| **Interaction during the interview**  (difficult passages) | ___________________________________________________________  ___________________________________________________________  ___________________________________________________________  ___________________________________________________________  ___________________________________________________________  ___________________________________________________________ |
| **Check** | Consent form signed? ☐  Questionnaire submitted? ☐    Interest in audio /transcript files? yes ☐ no ☐  Sent on: _______________________ |
