# Supplementary material for: Parental experience of the neuromotor development of children with congenital heart disease: an exploratory qualitative study
Source: BMC Pediatr. 2021 Oct 1;21:430. doi: 10.1186/s12887-021-02808-8 (PMC8485514; doi:10.1186/s12887-021-02808-8)
Supplement: Supplementary file 3 — Additional file 3. Examples of the methodological generation of themes. [file 12887_2021_2808_MOESM3_ESM.docx]

**Supplementary material III**

**Examples of the methodological generation of themes**

| **in vivo codes** | **subcategories** | **categories** | **themes** |
| --- | --- | --- | --- |
| *He always bottom-shuffles now; he never crawled.* | Leaving out  developmental steps | Child’s motor  development | burden |
| *It gets tiring. It is already tiring to have a baby, and then it is even more tiring to have a baby with needs* | Exhaustion | Parental physical and  psychological experience | burden |
| *I [mother of two children with CHD], I think physiotherapy is very important,*  *especially in the first year* | Physiotherapy | Support for child’s  motor development | need |
| *We should certainly have accepted more help, but not from the hospital, from specialists, but rather from the family* | Experience exchange with  family and equally affected | Communication | need |
